# Protective Effect of Total Flavonoids of Seabuckthorn (*Hippophae rhamnoides*) in Simulated High-Altitude Polycythemia in Rats

**DOI:** 10.3390/molecules171011585

**Published:** 2012-09-28

**Authors:** Ji-Yin Zhou, Shi-Wen Zhou, Xiao-Huang Du, Sheng-Ya Zeng

**Affiliations:** 1 Base for Drug Clinical Trial, Xinqiao Hospital, Third Military Medical University, Chongqing 400037, China; Email: zhoujiyin@gmail.com (J.-Y.Z.); zengshengya@sina.com (S.-Y.Z.); 2 Research Division, Southwest Hospital, Third Military Medical University, Chongqing 400038, China; Email: duxiaohuang@163.com

**Keywords:** total flavonoids of *Hippophae rhamnoides*, high-altitude polycythemia, hemodynamic parameters, hematologic parameters, erythropoietin

## Abstract

Seabuckthorn (*Hippophae rhamnoides* L.) has been used to treat high altitude diseases. The effects of five-week treatment with total flavonoids of seabuckthorn (35, 70, 140 mg/kg, ig) on cobalt chloride (5.5 mg/kg, ip)- and hypobaric chamber (simulating 5,000 m)-induced high-altitude polycythemia in rats were measured. Total flavonoids decreased red blood cell number, hemoglobin, hematocrit, mean corpuscular hemoglobin levels, span of red blood cell electrophoretic mobility, aggregation index of red blood cell, plasma viscosity, whole blood viscosity, and increased deformation index of red blood cell, erythropoietin level in serum. Total flavonoids increased pH, pO_2_, Sp_O2_, pCO_2_ levels in arterial blood, and increased Na^+^, HCO_3_^−^, Cl^−^, but decreased K^+^ concentrations. Total flavonoids increased mean arterial pressure, left ventricular systolic pressure, end-diastolic pressure, maximal rate of rise and decrease, decreased heart rate and protected right ventricle morphology. Changes in hemodynamic, hematologic parameters, and erythropoietin content suggest that administration of total flavonoids from seabuckthorn may be useful in the prevention of high altitude polycythaemia in rats.

## 1. Introduction

More than 140 million people live permanently at high altitude (>2,500 m) in North America, Central America, South America, East Africa, and Asia [1]. Furthermore, every year several hundreds of thousands of people from lowland areas move to higher altitudes for work or travel. High altitude hypoxia is a challenge for people residing in or visiting high altitudes. One of the health problems associated with life at high altitudes is chronic mountain sickness, also called Monge’s disease since it was first described in 1925 by Carlos Monge Medrano in Peru [2]. Exposure to high altitude is associated with an increase in red blood cell (RBC) production, which helps deliver oxygen to tissues more efficiently with mild polycythemia. As one subtype of chronic mountain sickness, high-altitude polycythemia (HAPC) shows increased blood viscosity and other adverse results caused by excessive erythrocytosis, with constitutional symptoms of headaches, confusion, insomnia, and bone pain [3,4]. The prominent role of hypoxia-induced erythropoietin increase in hypoxic erythrocytosis has been well established [5,6], but erythropoietin level does not correlate well with the amount of RBC production at high altitude [7]. Other possible factors related to HAPC include genetic adaptation, regulation of hypoxia-inducible factor-1, reactivity to erythropoietin, and delivery of iron [8,9].

Currently, therapeutic strategies for chronic mountain sickness are limited and include relocation to lower altitudes, repetitive bloodletting, and administration of drugs such as medroxyprogesterone, enalapril, almitrine, or acetazolamide [10]. However, no pharmacological or medical treatment is completely effective for curing HAPC [11], therefore, it is important to study plant extracts for HAPC treatment and other factors that could play a role in the prevention of chronic mountain sickness. A previous study reported that the seed oil of *Hippophae rhamnoides* L. (seabuckthorn) possesses protective activity against hypoxia and curtails hypoxia-induced enhanced vascular leakage in the brain [12].

Seabuckthorn, called “Shaji” in China, and naturally distributed over Asia and Europe, is a member of the *Elaeagnaceae* family. Seabuckthorn provides significant protection against hypoxia-induced pulmonary vascular leakage [13]. For its antioxidant, antiulcer, anticancer, antibacterial activities as well as cytoprotective, hepatoprotective, antihypertensive, cardio-protective properties and wound-healing activity [13,14,15,16,17], seabuckthorn has been used for treating diseases in Tibetan and Mongolian traditional medicines for a long time [18]. It is reported that in the high-performance liquid chromatograms of total flavonoids of seabuckthorn, 12 compounds have been identified, including quercetin 3-*O*-glucoside, isorhamnetin 3-*O*-rutinoside, quercetin, kaempferol, isorhamnetin [19]. With these as major constituents [20,21], total flavonoids have been demonstrated to be responsible for most of the bioactive properties of seabuckthorn. Our recent study showed that quercetin had protective potential on hypobaric hypoxia-induced damage through its regulation of cardiac function, arterial blood gases, antioxidants and nitric oxide metabolism [22]. The aim of the present study was to use a cobalt chloride- and hypobaric hypoxia-induced rat model to explore whether total flavonoids of seabuckthorn has protective effects on HAPC.

## 2. Results

### 2.1. Effect of Total Flavonoids on Routine Blood Parameters

Table 1 shows the results of hematological examinations. Five-week hypoxia and cobalt chloride exposure was associated with a significant increase in RBC, hemoglobin, hematocrit, and mean corpuscular hemoglobin levels compared with the normoxic rats. Total flavonoids (70, 140 mg/kg) and Tianji capsule (a Tibetan medicine compound preparation to treat altitude sickness) prevented these hypoxia- and cobalt chloride-induced changes approaching to those of the normoxic ones. But 35 mg/kg total flavonoids had no effect on hematologic parameters.

**Table 1:** Effect of total flavonoids of seabuckthorn on hematologic variables in high-altitude polycythemia rats.

Groups	RBC (×10^12^/L)	Hemoglobin (g/L)	Hematocrit (L/L)	MCH (pg)	Erythropoietin (mIU)
Normoxic control	7.14 ± 0.62	134.49 ± 13.33	0.392 ± 0.010	18.83 ± 0.94	7.05 ± 0.43
Hypoxic control	9.86 ± 0.54 ^*^	236.70 ± 13.09 ^*^	0.665 ± 0.013 ^*^	24.06 ± 1.50 ^*^	8.54 ± 0.25 ^*^
Hypoxic + 35 mg/kg TF	9.68 ± 0.83	231.49 ± 10.25	0.657 ± 0.014	24.02 ± 1.53	8.45 ± 0.31
Hypoxic + 70 mg/kg TF	8.28 ± 0.58 ^††^	184.52 ± 8.48 ^††^	0.518 ± 0.010 ^††^	22.36 ± 1.35 ^††^	7.51 ± 0.27 ^††^
Hypoxic + 140 mg/kg TF	8.13 ± 0.46 ^††^	174.45 ± 9.50 ^††^	0.479 ± 0.018 ^††^	21.50 ± 1.32 ^††^	7.42 ± 0.39 ^††^
Hypoxic + Tianji capsule	8.43 ± 0.55 ^††^	191.83 ± 10.49 ^††^	0.538 ± 0.010 ^††^	22.55 ± 1.59 ^†^	7.70 ± 0.15 ^††^

Values are means ± SD (*n* = 10). RBC: red blood cells; MCH: mean corpuscular hemoglobin. ^*^P < 0.01 compared with the normoxic control. ^†^*P* < 0.05, ^††^*P* < 0.01 compared with the hypoxic control.

### 2.2. Effect of Total Flavonoids on Serum Erythropoietin Content

Five-week hypoxia and cobalt chloride exposure significantly increased erythropoietin content in serum compared with the normoxic rats (Table 1). Total flavonoids (70, 140 mg/kg) and Tianji capsule prevented the hypoxia- and cobalt chloride-induced increased erythropoietin content approaching to that of the normoxic one. But 35 mg/kg total flavonoids had no effect on erythropoietin content.

### 2.3. Effect of Total Flavonoids on Hematologic Rheology Parameters

After five-week hypoxia and cobalt chloride exposure, span of RBC electrophoretic mobility, aggregation index of RBC, plasma viscosity, and whole blood viscosity at high/middle/low shear rate were significantly increased, and deformation index of RBC was significantly decreased (Table 2). Total flavonoids (70, 140 mg/kg) and Tianji capsule significantly decreased span of RBC electrophoretic mobility, aggregation index of RBC, plasma viscosity, whole blood viscosity and increased deformation index of RBC. But 35 mg/kg total flavonoids had no effect on these hypoxia- and cobalt chloride-induced changes.

**Table 2:** Effect of total flavonoids of seabuckthorn on haemorheology variables in high-altitude polycythemia rats.

Groups	Whole blood viscosity at			Plasma viscosity	RBC aggre-gation index	RBC deforma-tion index	RBC electro-phoretic time
	High shear rate (200/s)	Med. shear rate (30/s)	Low shear rate (3/s)				
Normoxic control	4.87 ± 0.90	6.09 ± 0.60	12.38 ± 1.20	1.44 ± 0.15	8.39 ± 0.85	0.70 ± 0.08	16.74 ± 0.95
Hypoxic control	15.80 ± 1.03 ^*^	16.27 ± 1.45 ^*^	29.06 ± 2.61 ^*^	2.23 ± 0.30 ^*^	16.17 ± 1.97 ^*^	0.48 ± 0.06 ^*^	28.28 ± 1.98 ^*^
Hypoxic + 35 mg/kg TF	15.66 ± 1.26	15.59 ± 1.14	28.79 ± 1.83	2.12 ± 0.31	16.09 ± 1.70	0.49 ± 0.07	28.16 ± 1.84
Hypoxic + 70 mg/kg TF	11.65 ± 0.91 ^†^	11.92 ± 1.82 ^†^	19.69 ± 2.17 ^†^	1.75 ± 0.14 ^†^	9.82 ± 0.74 ^†^	0.63 ± 0.09 ^†^	21.14 ± 1.41 ^†^
Hypoxic + 140 mg/kg TF	11.02 ± 1.17 ^†^	10.77 ± 1.43 ^†^	18.77 ± 2.50 ^†^	1.65 ± 0.14 ^†^	9.70 ± 1.17 ^†^	0.64 ± 0.09 ^†^	20.95 ± 1.24 ^†^
Hypoxic + Tianji capsule	11.83 ± 1.43 ^†^	11.07 ± 1.14 ^†^	20.94 ± 2.13 ^†^	1.72 ± 0.14 ^†^	10.35 ± 1.60 ^†^	0.62 ± 0.06 ^†^	21.21 ± 1.96 ^†^

Values are means ± SD (*n* = 10). ^*^P < 0.01 compared with the normoxic control. ^†^*P* < 0.01 compared with the hypoxic control.

### 2.4. Effect of Total Flavonoids on Hemodynamic Parameters

Hypoxia greatly decreased the mean arterial pressure, left ventricular systolic pressure, left ventricular end-diastolic pressure, left ventricular maximal rate of rise (+dP/d_tmax_), decrease (−dP/d_tmax_), but increased heart rate after five weeks (8 h per day) in a hypobaric, hypoxic chamber (simulating high altitude of 5,000 m). These decreases were partly improved and the increase was partly attenuated by total flavonoids (70, 140 mg/kg) and Tianji capsule, but not 35 mg/kg total flavonoids. A similar trend was seen in the relative increases in RV/(LV + S) ratio, and this increase was also partly attenuated by total flavonoids (70, 140 mg/kg) and Tianji capsule (Table 3). Animals exposed to five weeks of hypobaric hypoxia and cobalt chloride failed to gain body weight. Total flavonoids and Tianji capsule treatment during hypoxic exposure did not affect body weight (Data not shown).

**Table 3:** Effect of total flavonoids of seabuckthorn on hemodynamic variables in high-altitude polycythemia rats.

Groups	Left ventricle systolic pressure (mmHg)	Left ventricular end-diastolic pressure (mmHg)	Left ventricular +dp/dt (max)	Left ventricular −dp/dt (max)	Heart rate	Mean arterial pressure (mmHg)	RV/(LV + S)Ratio (%)
Normoxic control	172.387 ± 9.942	4.122 ± 0.335	18260.974 ± 1653.984	10574.945 ± 1086.160	388.0 ± 33.9	117.11 ± 8.43	0.34 ± 0.03
Hypoxic control	152.864 ± 8.646 ^*^	2.943 ± 0.319 ^*^	13502.003 ± 1361.975 ^*^	6980.370 ± 998.746^*^	441.0 ± 18.5^*^	97.15 ± 3.79^*^	0.48 ± 0.02^*^
Hypoxic + 35 mg/kg TF	154.924 ± 10.793	2.947 ± 0.386	13638.763 ± 909.229	7108.272 ± 867.963	438.0 ± 19.3	99.26 ± 4.90	0.45 ± 0.03
Hypoxic + 70 mg/kg TF	164.574 ± 9.250 ^†^	3.541 ± 0.535 ^††^	14663.368 ± 1112.082 ^†^	7813.614 ± 793.876 ^†^	391.0 ± 26.9 ^††^	105.77 ± 4.56 ^††^	0.41 ± 0.03 ^††^
Hypoxic + 140 mg/kg TF	166.387 ± 12.709 ^††^	3.614 ± 0.477 ^††^	15090.811 ± 887.559 ^††^	7906.700 ± 741.118 ^†^	387.0 ± 13.4 ^††^	108.56 ± 4.89 ^††^	0.40 ± 0.03 ^††^
Hypoxic + Tianji capsule	162.420 ± 8.740 ^†^	3.643 ± 0.542 ^††^	14796.312 ± 1261.311 ^†^	7927.825 ± 781.023 ^†^	396.0 ± 20.1 ^††^	103.10 ± 6.14 ^†^	0.41 ± 0.02 ^††^

Values are means ± SD (*n* = 10). ^*^P < 0.01 compared with the normoxic control. ^†^*P* < 0.05, ^††^*P* < 0.01 compared with the hypoxic control.

### 2.5. Effect of Total Flavonoids on Arterial Blood Gases

Five-week hypoxia and cobalt chloride exposure significantly decreased pH, pO_2_, Sp_O2_, and pCO_2_ in arterial blood compared with the normoxic rats. Total flavonoids (70, 140 mg/kg) and Tianji capsule all partly increased pH, pO_2_, Sp_O2_, and pCO_2_. But 35 mg/kg total flavonoids had no effect on blood gases variables (Table 4).

**Table 4:** Effect of total flavonoids of seabuckthorn on blood gases variables in high-altitude polycythemia rats.

Groups	pH	pO_2_ (mmHg)	Sp_O2_ (%)	pCO_2_ (mmHg)
Normoxic control	7.36 ± 0.03	121.8 ± 7.5	98.5 ± 0.5	35.6 ± 3.8
Hypoxic control	7.27 ± 0.03 ^*^	71.0 ± 4.0 ^*^	92.8 ± 2.3 ^*^	21.2 ± 2.0 ^*^
Hypoxic + 35 mg/kg TF	7.28 ± 0.01	74.1 ± 3.6	92.1 ± 2.1	21.3 ± 1.8
Hypoxic + 70 mg/kg TF	7.32 ± 0.02 ^††^	83.3 ± 4.4 ^††^	95.3 ± 0.9 ^††^	24.1 ± 1.0 ^††^
Hypoxic + 140 mg/kg TF	7.33 ± 0.04 ^††^	88.0 ± 3.3 ^††^	96.7 ± 1.2 ^††^	24.9 ± 1.9 ^††^
Hypoxic + Tianji capsule	7.33 ± 0.04 ^††^	82.4 ± 3.4 ^††^	94.2 ± 1.3 ^†^	23.5 ± 1.6 ^†^

Values are means ± SD (*n* = 10). ^*^P < 0.01 compared with the normoxic control. ^†^*P* < 0.05, ^††^*P* < 0.01 compared with the hypoxic control.

### 2.6. Effect of Total Flavonoids on Electrolyte Concentration

Table 5 shows the results of hematological examinations. Five-week hypoxia and cobalt chloride exposure significantly increased blood K^+^ concentration, but decreased Na^+^, HCO_3_^−^, and Cl^−^ concentrations compared with the normoxic rats. There was also a decline on pH in rats with high-altitude polycythemia (Table 4). Total flavonoids (70, 140 mg/kg) and Tianji capsule prevented these hypoxia- and cobalt chloride-induced changes approaching to those of the normoxic ones, but 35 mg/kg total flavonoids had no effect on blood electrolytes.

**Table 5:** Effect of total flavonoids of seabuckthorn on blood electrolyte variables in high-altitude polycythemia rats.

Groups	Na^+^ (mmol/L)	K^+^ (mmol/L)	Cl^−^ (mmol/L)	HCO_3_^−^ (mmol/L)
Normoxic control	135.6 ± 3.3	4.97 ± 0.41	104.2 ± 2.7	18.1 ± 1.6
Hypoxic control	117.3 ± 3.0 ^*^	6.03 ± 0.25 ^*^	82.9 ± 2.1 ^*^	11.4 ± 1.3 ^*^
Hypoxic + 35 mg/kg TF	118.4 ± 2.9	5.87 ± 0.30	84.4 ± 2.8	11.5 ± 1.1
Hypoxic + 70 mg/kg TF	130.8 ± 1.2 ^†^	5.23 ± 0.25 ^†^	102.6 ± 2.1 ^†^	12.9 ± 1.3 ^†^
Hypoxic + 140 mg/kg TF	131.7 ± 1.8 ^†^	5.26 ± 0.23 ^†^	103.8 ± 2.2 ^†^	13.8 ± 1.2 ^†^
Hypoxic + Tianji capsule	128.6 ± 2.5 ^†^	5.14 ± 0.18 ^†^	101.9 ± 1.4 ^†^	13.2 ± 0.8 ^†^

Values are means ± SD (*n* = 10). ^*^P < 0.01 compared with the normoxic control. ^†^*P* < 0.01 compared with the hypoxic control.

### 2.7. Effect of Total Flavonoids on Right Ventricle Morphology

**Figure 1:**
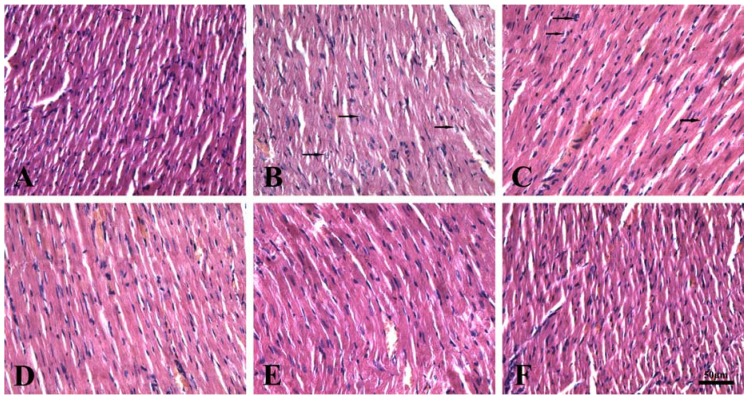
Effect of total flavonoids of seabuckthorn on HE staining on right ventricle of heart. (**A**) normoxic control group, (**B**) hypoxic control group, (**C**) hypoxic treated with total flavonoids (35 mg/kg) group, (**D**) hypoxic treated with total flavonoids (70 mg/kg) group, (**E**) hypoxic treated with total flavonoids (140 mg/kg) group, (**F**) hypoxic treated with Tianji capsule (500 mg/kg) group. The black arrow represents granular degeneration. Magnification: ×400.

The typical HE staining results obtained upon histological examination of right ventricle are shown in Figure 1. Normal rats showed no significant pathological structural changes (Figure 1A). Disordered myocardial fibers and myocardial cell granular degeneration in the right ventricle of the hypobaric hypoxia model group (Figure 1B) were significantly different compared with the control group (Figure 1A). More homogeneous myocardial fiber arrangement and occasionally granular degeneration of myocardial cells were shown in 70, 140 mg/kg total flavonoids (Figure 1D,E) and Tianji capsule (Figure 1F) groups compared with model group. While 35 mg/kg total flavonoids (Figure 1C) could not improve the chronic hypoxia-induced pathological changes in right ventricle.

## 3. Discussion

Five-week treatment with total flavonoids of seabuckthorn improved hemodynamic, hematologic parameters, and erythropoietin content, which indicates that it is useful in the prevention of cobalt chloride- and hypobaric chamber-induced high-altitude polycythemia in rats. This study investigated for the first time the effect of total flavonoids of seabuckthorn on high-altitude polycythemia in rats. Total flavonoids improved hematologic parameters which may result in a reduction in erythropoiesis and a minimization of the changes in the right heart, but the exact mechanism/s of action of the total flavonoids on high-altitude polycythemia still needs a considerable amount of work in the future.

Total flavonoids of seabuckthorn contain 12 compounds, with the major constituents being quercetin, isorhamnetin, and kaempferol [20,21]. Because quercetin has significant antioxidant activity with broad uses [23], and furthermore, our recent study showed that quercetin had protective potential on hypobaric hypoxia-induced damage [22], it may be responsible for the preventive effect of total flavonoids on high-altitude polycythemia in rats. According to our preliminary experiments (data not shown) and some studies using three doses of total flavonoids of seabuckthorn at 50, 100 and 150 mg/kg [24,25], this study used three doses of total flavonoid at 35, 70 and 140 mg/kg.

This cobalt chloride- and hypobaric hypoxia-induced rat model has been widely used by our laboratory and other laboratories to research the effects of high-altitude hypoxia on the body. The decompression treatment did not obviously exacerbate the severity of the high-altitude effects on rats. No signs of high-altitude sicknesses such as cerebral or pulmonary edema were observed in these rats. Cobalt chloride is used widely as a reagent to mimic hypoxia in both *in vitro* and *in vivo* studies [26]. As in the human disease, the rat model of human chronic mountain sickness manifests excessive polycythemia and hypervolemia [27].

Our results show that total flavonoids decreased RBC, hemoglobin, hematocrit, mean corpuscular hemoglobin levels, span of RBC electrophoretic mobility, aggregation index of RBC, plasma viscosity, whole blood viscosity, and increased deformation index of RBC. In patients with chronic mountain sickness, accentuated hypoxemia is believed to result from hypoventilation, even though hypoventilation has not been observed consistently. Impaired pulmonary gas exchange is found in patients with chronic mountain sickness and must contribute to systemic arterial hypoxemia [28]. Accentuated hypoxemia in rats during hypoxic exposure also resulted from abnormal gas exchange [29,30]. Total flavonoids also increased pH, pO_2_, Sp_O2_, and pCO_2_ levels in arterial blood. pCO_2_ fell in response to hypoxic exposure to comparable levels in rat [31], indicative of a comparable alveolar hyperventilation. Suppression of the development of pulmonary hypertension in rats during hypoxic exposure was associated with an increased pO_2_ and an attenuated polycythemic response without any change in ventilation. In our results, high-altitude polycythemia rats showed increased blood K^+^ concentration, but decreased Na^+^, HCO_3_^−^, Cl^−^ concentrations and pH. Total flavonoids prevented these hypoxia- and cobalt chloride-induced changes of blood electrolytes. High-altitude exposure and chronic hypoxia can lead to blood electrolytes disturbance [32,33].

Our results also show that total flavonoids decreased serum erythropoietin level in high-altitude polycythemia rats. The early finding that a humoral erythropoiesis-stimulating factor increased rapidly on hypoxic exposure and declined toward control levels led to the notion of a negative feedback control mechanism regulating erythropoiesis. According to this concept, hypoxia stimulates erythropoietin production and erythropoiesis; the resulting polycythemia increases the O_2_-carrying capacity and O_2_ content of blood, and this improves tissue oxygenation and turns off further production of erythropoietin [34]. The idea of erythropoietin-O_2_-carrying capacity feedback is inherent in erythropoietin dose-response curves published [35]. Nevertheless, a negative-feedback control mechanism does not account for the regulation of the polycythemic response to environmental hypoxia [36]. Consequently, the typical signs of HAPC develop as follows: profound arterial hypoxemia, persistent production of erythropoietin, and then extraordinary polycythemia. The above gained results suggest that total flavonoids may also have beneficial effects on high-altitude related and polycythemia related diseases.

## 4. Experimental

### 4.1. Reagents

Total flavonoids of seabuckthorn were provided by Baoji F.S. Biological Development Co. Ltd., Baoji, China. Cobalt chloride (CoCl_2_) was brought from Beijing Hengye Zhongyuan Chemical Co., Ltd., Beijing, China. The kits of K^+^, Na^+^, and Cl^−^ were obtained from Nanjing Jiancheng Bioengineering Institute, Nanjing, China. The erythropoietin kit was provided by Beijing Zhongshan Golden Bridge Biotechnology Co. Ltd, Beijing, China. Tianji capsules were brought from Chengdu Baicao in development and application of Chinese medicine Co. Ltd, Chengdu, China.

### 4.2. Experimental Procedure

Male Wistar rats (180–220 g) were provided by the Third Military Medical University, Chongqing, China. A total of 60 rats were randomly placed into six groups (10 rats in each group): normoxic (normal control) group, a hypoxic (model control) group, three different doses of total flavonoids-treated groups, and a Tianji capsule-treated (positive control) group. High-altitude polycythemia in rats were induced by cobalt chloride (ip) and hypobaric chamber. Hypoxic rats were raised in a hypobaric chamber, where atmospheric pressure was reduced to simulate a high altitude of 5,000 m [37]. The partial pressure of nitrogen fell as total pressure declines on ascent, but nitrogen percentage did not change in the atmosphere. The ascending speed and descending speed for the hypobaric chamber were limited to 4–5 m/s. Normal control rats were raised at an altitude of 300 m out of the hypobaric chamber. Before being placed in the chamber, rats in the four treated groups were injected cobalt chloride (5.5 mg/kg, ip) and given total flavonoids (35, 70, or 140 mg/kg, ig) and Tianji capsule (500 mg/kg) per day. Rats in model control group were injected cobalt chloride and received the same volume of normal saline. Normal control rats were injected and received the same volume of normal saline. After 12 h hypoxic exposure, hypoxic rats were returned to normobaric condition. Animals were continuously treated for five weeks. The animals were housed in community cages and maintained under regular laboratory conditions (25 ± 2 °C, 12 h light-dark cycle, free access to water and standard rodent chow). All experiments were performed with the approval of the Animal Studies Ethics Committee of Xinqiao Hospital, Third Military Medical University.

### 4.3. Hemodynamic Measurements

At the end of the exposure period, animals were weighed and anesthetized with urethane (1 g/kg, ip). Mean arterial pressure, heart rate, left ventricular systolic pressure, left ventricular end-diastolic pressure, left ventricular maximal rate of rise (+dP/d_tmax_) and decrease (−dP/d_tmax_) were measured using a tube introduced into the right external carotid artery by a cut-down in the neck and advanced to the left ventricle. The catheters were linked to a pressure transducer that was connected to a multichannel physiological recorder PowerLab ML870/P (AD Instruments, Bella Vista, NSW, Australia). After a 10 min stabilization period, the measurements were recorded at a sampling rate of 50 Hz and stored digitally [22,38].

### 4.4. Hematologic Measurements

Arterial blood gas samples were obtained anaerobically by withdrawing 1 mL of blood from the rat via the right external jugular vein for analysis. pO_2_, pCO_2_, pH, and HCO_3_^−^ levels were calculated by microelectrodes at 37 °C (Model ALB5 blood gas analyzer, Radiometer, Copenhagen, Denmark). After the hemodynamic investigation, the rats were sacrificed by exsanguinations via the right external jugular vein. Parts of collected blood samples were used for measurement of K^+^, Na^+^, and Cl^−^ levels by commercial kit as the manufacturer’s instructions. Serum erythropoietin was measured by radioimmunoassay using a commercial kit. A LBY-N6B hemorheology auto-analyzing instrument (Beijing Precil Instrument Co. Ltd., Beijing, China) was used to measure haemorheology parameters, such as plasma viscosity, whole blood viscosity at high/middle/low shear rate, span of RBC electrophoretic mobility and aggregation index of RBC. Biocode Hycel Celly 70 Hematology Analyzer (Biocode Hycel, Rennes, France) was used to measure RBC, hemoglobin, hematocrit and mean corpuscular hemoglobin (MCH).

### 4.5. Determination of Heart Weight

The heart was removed, the chambers were opened, and then the heart was weighed after excess blood had been absorbed with filter paper. The atria and large blood vessels were then removed. The heart was divided into two pieces: the right ventricular free wall (RV) and the remaining piece, comprising the left ventricle (LV) and septum. Each piece was weighed. The right ventricle mass index was defined as the weight ratio between the RV and the LV plus septum [RV/(LV + S)] [39].

### 4.6. Histology

Heart tissues were fixed by immersion in 10% buffered formalin and embedded in paraffin for histological analyses. Seven-μm sections were stained with hematoxylin and eosin staining. The samples were coded and the slides were sent for light microscopic examination by a researcher who was unaware of the specimen identity.

### 4.7. Statistical Analysis

Results were expressed as mean ± SD. Significant differences were established by one-way ANOVA using SPSS 13.0 [40], followed by the least significant difference multiple comparisons test. Differences were considered significant at *P* < 0.05.

## 5. Conclusions

Our data collectively show that after five-week treatment with total flavonoids of seabuckthorn, RBC, hemoglobin, hematocrit, mean corpuscular hemoglobin levels, span of RBC electrophoretic mobility, aggregation index of RBC, plasma viscosity, whole blood viscosity were significantly decreased, and deformation index of RBC and erythropoietin in serum and heart was decreased; pH, Pa_O2_, Sp_O2_, Pa_CO2_ levels were partly increased, and Na^+^, HCO^−^, Cl^−^ concentrations were increased, but K^+^ was decreased; partly increased mean arterial pressure, left ventricular systolic pressure, left ventricular end-diastolic pressure, left ventricular maximal rate of rise (+dP/d_tmax_) and decrease (−dP/d_tmax_), and partly decreased heart rate; right ventricle morphology was improved. Our results suggest that the effects of total flavonoids on hemodynamic, hematologic parameters, erythropoietin content may be related to its protective potential on HAPC.
